# Transbronchial Lung Cryobiopsy and Awake Video-Assisted Thoracic Surgery in Interstitial Lung Disease: Complementary Roles in a Stepwise Diagnostic Approach

**DOI:** 10.3390/diagnostics16132095

**Published:** 2026-07-03

**Authors:** Umberto Masi, Alessandro Sanduzzi Zamparelli, Stefano Sanduzzi Zamparelli

**Affiliations:** 1 Interventional Pulmonology Unit, A. Cardarelli Hospital, 80131 Naples, Italy; 2 Department of Clinical Medicine and Surgery, University of Naples “Federico II”, 80131 Naples, Italy; sanduzzi@unina.it; 3 UNESCO Chair for Health Education and Sustainable Development, University of Naples “Federico II”, 80131 Naples, Italy; 4 ERN Lung, 60596 Frankfurt am Main, Germany; 5 Division of Pneumology and Semi-Intensive Respiratory Therapy, A. Cardarelli Hospital, 80131 Naples, Italy

**Keywords:** interstitial lung disease, transbronchial lung cryobiopsy, awake video-assisted thoracoscopic surgery, diagnostic strategy, multidisciplinary discussion

## Abstract

The diagnostic evaluation of interstitial lung diseases (ILDs) remains challenging when clinical assessment and imaging findings are inconclusive. Although surgical lung biopsy has traditionally represented the diagnostic gold standard, its invasiveness and perioperative risks limit its applicability, particularly in patients with advanced disease or impaired respiratory reserve. This review aims to examine the evolving roles of transbronchial lung cryobiopsy (TBLC) and awake video-assisted thoracoscopic surgery (Awake VATS) within contemporary diagnostic pathways for ILD. A narrative review of the current literature was performed, focusing on studies evaluating the diagnostic performance, safety profiles, clinical indications, and complementary integration of TBLC and Awake VATS in patients with suspected ILD. Evidence from multidisciplinary ILD referral centers and recent guideline recommendations was critically analyzed. TBLC has progressively emerged as an appropriate first-line histological procedure in many ILD centers, providing a pooled diagnostic yield of approximately 80% with an acceptable safety profile. Awake VATS has refined the surgical approach by preserving spontaneous ventilation while maintaining high diagnostic accuracy. Current evidence suggests that these techniques should be considered complementary rather than competitive. A TBLC-first strategy, followed by selective surgical escalation when endoscopic sampling is non-diagnostic or insufficient, appears to achieve diagnostic accuracy comparable to upfront surgical biopsy while reducing complications, length of hospital stay, and overall patient burden. The choice between Awake VATS and conventional surgical biopsy should be individualized according to patient characteristics, institutional expertise, and available resources. TBLC and Awake VATS represent complementary tools within a multidisciplinary, personalized, and risk-adapted diagnostic framework for ILD. Their integrated use enables optimization of diagnostic accuracy while minimizing procedural invasiveness and improving patient safety, supporting a stratified approach to histological assessment in contemporary clinical practice.

## 1. Introduction

Interstitial lung diseases (ILDs) comprise a broad and heterogeneous group of conditions characterized by inflammation, fibrosis, and remodeling of the pulmonary parenchyma, leading to progressive impairment of respiratory function. Despite significant advances in disease classification and imaging techniques, making a confident diagnosis remains challenging in a not-negligible proportion of patients. In such contexts, making an accurate diagnosis is not merely a classificatory exercise, but rather a fundamental prerequisite for appropriate prognostic stratification and the implementation of effective therapeutic strategies [1,2]. The widespread use of high-resolution computed tomography (HRCT) has significantly reshaped the diagnostic approach to ILD, allowing identification of imaging patterns that may obviate the need for histological confirmation in selected cases, particularly in idiopathic pulmonary fibrosis (IPF) [3,4,5]. Nevertheless, many patients show indeterminate or probable patterns, or features suggestive of alternative diagnoses that cannot be conclusively defined through imaging alone. In such cases, diagnostic uncertainty frequently persists despite structured multidisciplinary discussion (MDD), thereby necessitating histological sampling [6]. Surgical lung biopsy performed via video-assisted thoracoscopic surgery (VATS) has historically been regarded as the gold standard for histological diagnosis of ILD, owing to its ability to provide large, representative tissue specimens, including subpleural regions [7]. However, the invasiveness of the procedure, the requirement for general anesthesia with single-lung ventilation, and the non-negligible risk of perioperative complications have limited its applicability, particularly in a patient population that is frequently older, frail, and characterized by reduced respiratory reserve [8,9,10]. These limitations have driven the search for alternative diagnostic strategies capable of maintaining adequate diagnostic accuracy while reducing procedural burden.

Within this context, transbronchial lung cryobiopsy (TBLC) has emerged as an endoscopic technique capable of obtaining lung parenchymal samples that are significantly larger and better preserved than those obtained with conventional transbronchial forceps biopsy. Over the past decade, TBLC has evolved from an experimental procedure to a widely adopted tool in ILD referral centers, supported by a growing body of observational studies, randomized trials, meta-analyses, and prospective trials [11,12]. Although its diagnostic yield remains lower than that of surgical biopsy, TBLC offers a more favorable safety profile, reduced invasiveness, and a substantial reduction in hospital length of stay and overall costs. In parallel, thoracoscopic surgery performed under spontaneous ventilation, known as awake video-assisted thoracoscopic surgery (Awake VATS), has emerged as an evolution of conventional surgical biopsy. This technique aims to preserve respiratory physiology by avoiding endotracheal intubation and mechanical ventilation, both of which are recognized as risk factors in patients with ILD. Awake VATS allows acquisition of high-quality surgical specimens with a reduced anesthetic burden, representing an intermediate option between TBLC and conventional VATS [13,14,15].

The accumulating body of evidence has progressively clarified that TBLC and Awake VATS should not be viewed as competing techniques, but rather as complementary tools within a stratified diagnostic pathway. Recent studies, including randomized controlled trials, have demonstrated that a sequential approach, based on initial TBLC followed by surgical biopsy only in non-diagnostic cases, achieves a final diagnostic accuracy comparable to that of upfront surgery, while significantly reducing complication rates and overall clinical burden [16,17].

**Figure 1:**
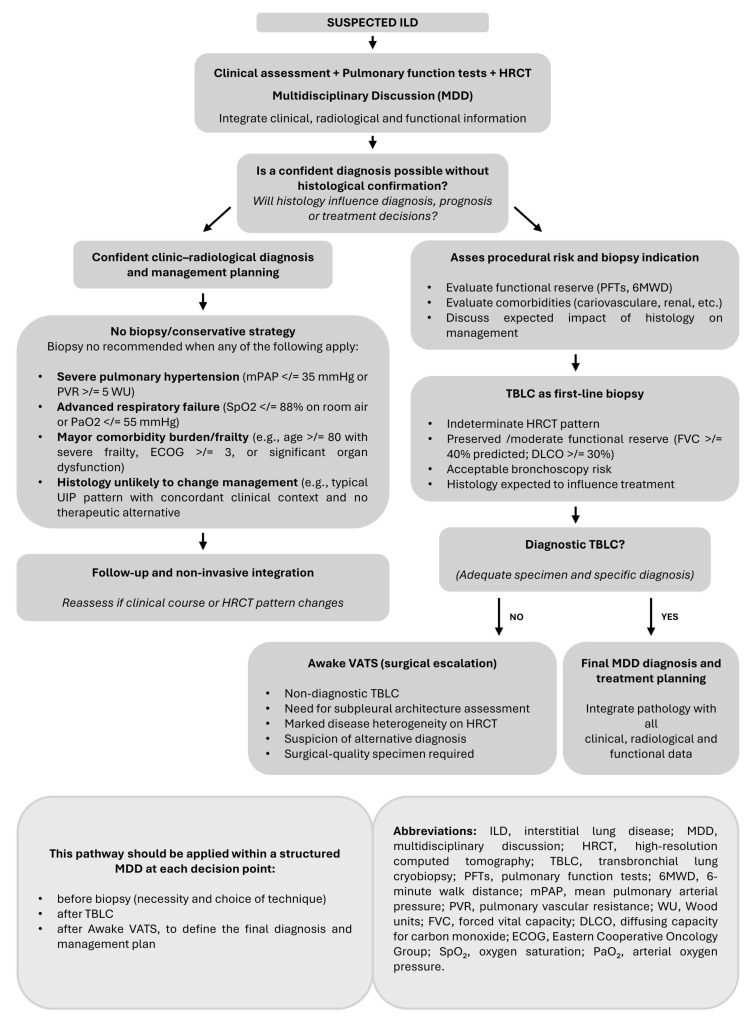
Proposed multidisciplinary step-up diagnostic pathway for patients with suspected interstitial lung disease (ILD).

In light of these developments, a critical and integrated appraisal of the available evidence is warranted, shifting the focus from isolated comparisons of diagnostic techniques to their complementary roles within a multidisciplinary, risk-adapted diagnostic framework. Accordingly, this narrative review synthesizes evidence from randomized controlled trials, meta-analyses, prospective studies, and selected clinically relevant observational series to delineate the roles of TBLC and Awake VATS within a multidisciplinary, risk-adapted diagnostic framework. Importantly, the proposed pathway should not be interpreted as proof that TBLC followed specifically by Awake VATS has been definitively validated in all settings. Rather, current evidence supports a TBLC-first strategy with selective surgical escalation when histology remains necessary, while Awake VATS may be considered a less invasive surgical option in experienced centers [11,15,16,17] (Figure 1).

Histological sampling should be considered only when expected to influence diagnosis, prognosis, or treatment decisions. Multidisciplinary discussion (MDD) is central before biopsy to determine whether tissue sampling is necessary, to select the most appropriate biopsy technique and sampling site, and after TBLC or surgical biopsy to integrate histopathological findings with clinical, radiological, and functional data. TBLC is considered the preferred initial histological procedure in appropriate candidates, whereas surgical biopsy (Awake VATS or conventional VATS according to patient characteristics, institutional expertise, and local resources) is reserved for selected patients with non-diagnostic TBLC or when larger, subpleural specimens are required. Patients in whom biopsy is unlikely to modify management or who present prohibitive procedural risk should be managed through multidisciplinary clinical–radiological assessment and follow-up.

## 2. Materials and Methods

This narrative review was conducted to provide a clinically oriented synthesis of the evidence regarding transbronchial lung cryobiopsy, awake video-assisted thoracoscopic surgery, and their potential integration within a stepwise diagnostic strategy for interstitial lung disease. A literature search was performed in PubMed, covering studies published from January 2010 to March 2026. Search terms included combinations of “interstitial lung disease”, “transbronchial lung cryobiopsy”, “cryobiopsy”, “surgical lung biopsy”, “video-assisted thoracoscopic surgery”, “awake VATS”, “non-intubated thoracic surgery”, “spontaneous ventilation thoracic surgery”, “multidisciplinary discussion”, and “diagnostic pathway”. Reference lists of relevant articles were also screened to identify additional studies of potential interest. The review considered included randomized controlled trials, prospective and retrospective observational studies, systematic reviews, meta-analyses, consensus documents, and clinically relevant technical reports. Because this was a narrative review rather than a systematic review, no formal risk-of-bias assessment or meta-analysis was performed. Studies summarized in Table 1 were selected because they were considered representative of the available evidence according to study design, sample size, clinical relevance, and contribution to the proposed diagnostic framework.

## 3. Clinical and Technical Background

Histological evaluation is not required in all patients with interstitial lung disease, as integration of clinical data, pulmonary function testing, high-resolution computed tomography, and multidisciplinary discussion is often sufficient to establish a confident diagnosis [6,25]. Lung biopsy becomes essential when imaging findings are indeterminate or when alternative diagnoses, such as hypersensitivity pneumonitis, rare ILDs, or connective tissue disease-associated ILD, cannot be reliably established based on clinical and radiological data alone, and histological evaluation is expected to inform diagnosis, prognosis, or treatment [5,17,26,27].

Biopsy should be considered only when histological information is expected to modify diagnosis, prognosis, treatment choice, or eligibility for disease-specific therapy. Conversely, biopsy may be avoided when HRCT and clinical features already provide sufficient diagnostic confidence, when histology is unlikely to change management, or when procedural risk clearly outweighs potential benefit. Examples include severe resting hypoxemia, advanced respiratory failure, severe pulmonary hypertension, unstable cardiovascular disease, major frailty, or extensive comorbidity burden [7,8,9,10,25].

MDD should therefore occur before any biopsy is planned. At this stage, MDD defines whether tissue is necessary, which technique is most appropriate, and which lung areas should be sampled. After TBLC, MDD should reassess diagnostic confidence and determine whether surgical biopsy is still required. After Awake VATS or conventional surgical biopsy, MDD remains essential to integrate histology with clinical, functional, and radiological data.

From a pathological perspective, the diagnosis of ILD requires assessment that extends beyond the identification of isolated cellular or fibrotic features. Pathologists need specimens that preserve lung architecture and ideally include subpleural regions, fibrotic areas alternating with relatively preserved parenchyma, the presence or absence of fibroblastic foci, inflammatory infiltrates, granulomas, and the patchy distribution of fibrosis. These architectural features are essential for differentiating histological patterns and for providing diagnostically meaningful input within MDD [6,11,28,29]. Conventional transbronchial forceps biopsies are often inadequate in this regard, as they yield small, frequently crushed specimens lacking architectural continuity [11,30,31]. TBLC, in contrast, provides better preservation of tissue architecture, although it does not consistently reach subpleural regions [11]. This advantage derives from the use of a cryoprobe that rapidly freezes lung parenchyma through expansion of an internal refrigerant gas, enabling extraction of tissue with minimal mechanical distortion [32]. As a result, TBLC yields larger specimens than forceps biopsy, typically measuring 5–7 mm in diameter compared with 1–2 mm, thereby enhancing histopathological interpretability and diagnostic confidence [31,33].

Awake video-assisted thoracoscopic surgery, in turn, allows acquisition of complete surgical specimens that include peripheral and subpleural lung tissue. This technique represents an evolution of conventional thoracoscopic surgery aimed at reducing anesthetic and respiratory burden in patients with ILD by preserving spontaneous ventilation and avoiding endotracheal intubation and mechanical ventilation [15,34]. Performed under locoregional anesthesia with light sedation, Awake VATS maintains respiratory physiology while enabling collection of large, highly representative lung samples [35,36].

Taken together, these considerations indicate that no single biopsy technique can be regarded as intrinsically superior; rather, the optimal approach should be selected according to the specific clinical context and the type of histological information required to support an accurate and meaningful diagnosis [11,15,16,17,34] (Table 2).

## 4. Transbronchial Lung Cryobiopsy: Consolidated Evidence and Recent Applications

TBLC represents one of the most significant advances in the histological diagnosis of ILD over the past two decades, as a minimally invasive procedure positioned between conventional transbronchial forceps biopsy and surgical lung biopsy. By providing larger and better-preserved parenchymal specimens through a minimally invasive endoscopic approach, TBLC has substantially reduced the need for upfront surgical biopsy while maintaining meaningful histopathological yield, leading to its widespread adoption in specialized ILD referral centers [11,37,38].

The strongest evidence supporting TBLC derives from a large meta-analysis of over 6000 patients, which reported a pooled diagnostic yield of approximately 81% [38]. Nevertheless, the high statistical heterogeneity observed reflects substantial variability among the included studies, suggesting that TBLC should not be regarded as a uniformly standardized procedure but rather as a technique highly dependent on clinical, technical, and organizational factors [38,39]. Indeed, analysis of determinants of diagnostic yield demonstrated that several variables were significantly associated with an increased likelihood of achieving a meaningful diagnosis, including the use of general anesthesia, selection of the biopsy site through a pre-procedural MDD, the use of larger-diameter cryoprobes, and better baseline respiratory functional reserve, as reflected by higher forced vital capacity and diffusing capacity for carbon monoxide [20,38].

These findings indicate that TBLC is not merely a technical maneuver but a complex diagnostic process requiring careful planning, multidisciplinary integration, and an appropriate operational setting [11]. In skilled referral centers, diagnostic yields frequently approach or exceed 90%, underscoring the importance of standardization and operator expertise [20,40].

From a safety perspective, TBLC demonstrates a favorable profile compared with surgical lung biopsy [23,38,39]. Pneumothorax represents the most frequently reported complication, with an overall incidence of approximately 5%, while moderate-to-severe bleeding occurs in about 12% of cases [39]. Although these events are not negligible, the majority of complications can be managed conservatively or endoscopically, and the need for rescue surgical intervention is rare. Procedure-related mortality is exceedingly uncommon, confirming that when performed by skilled teams in appropriately equipped environments, TBLC exhibits a favorable safety profile that is markedly superior to that of traditional surgical lung biopsy [11,23,38,39].

Subsequent prospective studies and randomized trials have refined TBLC technique, particularly through the introduction of next-generation smaller-diameter cryoprobes. While early 2.4 mm probes yielded excellent specimen quality at the cost of slightly higher complication rates, 1.9 mm probes have become the current standard by offering an optimal balance between diagnostic yield and safety. More recent disposable probes (1.7 mm and 1.1 mm) have demonstrated comparable diagnostic performance with potential reductions in bleeding risk, although increased pneumothorax rates have been reported in some studies, likely due to more peripheral sampling [18,20,21]. Notably, although specimen surface area is reduced, tissue weight appears to be similar, suggesting that increased biopsy depth may compensate for reduced lateral dimensions.

Technological innovations, such as the integration of electromagnetic navigation systems, have further enhanced TBLC performance. Traditionally, fluoroscopy served as the primary guidance modality, but it has inherent limitations in targeting precision, particularly in cases with difficult-to-reach areas or heterogeneous disease distribution. Electromagnetic navigation enables three-dimensional bronchial pathway planning and more accurate access to predefined target segments. Recent studies have shown that, although navigation does not consistently translate into a higher overall diagnostic yield, it significantly increases the likelihood that the obtained specimen meaningfully contributes to the final diagnosis by improving tissue representativeness [19]. This advantage is particularly relevant in complex cases, where non-representative sampling may lead to non-diagnostic or equivocal results.

Procedural planning remains central to TBLC success [11,23]. Careful selection of the biopsy sites, ideally guided by pre-procedural MDD, allows avoidance of end-stage fibrotic regions and prioritization of transitional areas, maximizing diagnostic yield while minimizing risk. Similarly, the anesthetic setting plays a key role: although TBLC can be performed under deep sedation, numerous studies indicate that general anesthesia, in appropriately selected patients, combined with enhanced airway protection and more effective bleeding control using endobronchial balloon blockers, is associated with superior safety and specimen quality [11,20,23].

Analyses of complications have identified key risk factors, with pneumothorax more common in patients with advanced fibrosis, reduced diffusing capacity, peripheral or upper-lobe biopsies, while bleeding risk is primarily related to cryoprobe diameter, biopsy site, and pulmonary hypertension [11,20,23] (Table 3). Recognition of these factors may improve patient selection and procedural refinement, enhancing the overall safety of TBLC.

Beyond procedural considerations, TBLC has led to a significant organizational impact, reducing reliance on upfront surgical biopsy, shortening diagnostic timelines, and lowering healthcare costs. Its implementation has strengthened multidisciplinary collaboration, reduced unnecessary surgical procedures and improved diagnostic efficiency within ILD referral centers [11,17].

Overall, the accumulated evidence supports TBLC as an appropriate first-line histological procedure in the diagnostic workup of ILD [11,38]. When performed within a structured, multidisciplinary framework and by experienced operators, TBLC offers a favorable balance between diagnostic yield and safety, with ongoing innovations likely to further consolidate its role and reduce the need for primary surgical lung biopsy [17,23].

## 5. Spontaneous Ventilation Thoracoscopic Surgery (Awake VATS): Rationale, Technique, and Evidence

The evidence base for Awake VATS in ILD is more limited and heterogeneous than that available for TBLC. Several studies derive from ILD-specific cohorts, whereas other procedural and anesthesiologic considerations are extrapolated from the broader literature on non-intubated thoracic surgery. This distinction is important, because ILD patients have specific risks related to reduced lung compliance, impaired gas exchange, pulmonary hypertension, and susceptibility to acute exacerbation.

Spontaneous ventilation thoracoscopic surgery, commonly referred to as Awake VATS, represents a relevant evolution of surgical lung biopsy, aimed at reducing the physiological burden of mechanical ventilation in ILD patients, particularly those with advanced fibrosis or limited respiratory reserve. By preserving spontaneous ventilation and avoiding endotracheal intubation and single-lung ventilation, the cornerstone of conventional VATS, Awake VATS minimizes ventilator-induced mechanical and inflammatory injury while maintaining access to large and representative lung specimens [15,34,35,41].

The pathophysiological rationale of Awake VATS is grounded in avoiding the mechanical and inflammatory effects of artificial ventilation on structurally compromised lung parenchyma. In ILD, reduced lung compliance, heterogeneous ventilation, and mechanically fragile fibrotic regions increase susceptibility to ventilator-induced lung injury. By eliminating endotracheal intubation and single-lung ventilation, Awake VATS reduces the risk of volutrauma, atelectrauma, and barotrauma. During the procedure, passive and gradual collapse of the operative lung occurs through loss of pleural pressure, while the contralateral lung continues spontaneous ventilation, resulting in greater respiratory stability compared with conventional surgery [15,41].

Technically, Awake VATS is performed under light-to-moderate sedation, often using agents such as dexmedetomidine, which allows for cooperative sedation while preserving spontaneous respiration. Pain control is achieved through locoregional anesthesia, including paravertebral or intercostal nerve blocks, providing effective analgesia without impairing respiratory function [42,43]. Thoracoscopic access, either uniportal or multiportal depending on expertise, enables targeted subpleural biopsies guided by preoperative imaging. The specimens obtained are substantially larger than those achieved with TBLC and preserve full parenchymal architecture, making Awake VATS particularly valuable when comprehensive histological assessment is required [34,35].

Patient selection remains crucial. Awake VATS may be particularly valuable when surgical-quality tissue is required, when subpleural architecture is essential, when disease distribution is markedly heterogeneous, or when TBLC is non-diagnostic. Conversely, caution is warranted in patients with severe pulmonary hypertension, unstable respiratory failure, inability to cooperate, extensive pleural adhesions, or other conditions that may compromise procedural safety. Successful implementation also depends on the availability of experienced thoracic surgeons, dedicated anesthesiology support, and institutional readiness for conversion to conventional intubated surgery if required.

Evidence supporting Awake VATS has grown steadily, culminating in a recent meta-analysis of thirteen studies (2010–2023) including 675 patients. Reported diagnostic yield ranged from 85% to 100%, with consistently excellent specimen quality and no reported 30-day mortality [24]. Overall complication rates were below 10% and were predominantly minor, with postoperative air leak being the most frequent event. In indirect comparisons with conventional VATS, Awake VATS demonstrated a significantly lower overall complication rate, while maintaining equivalent diagnostic yield, suggesting a safety advantage in ILD populations. These findings suggest that Awake VATS offers a safety advantage over conventional surgery by providing surgical-quality tissue without intubation, particularly in fragile patients or those with significant comorbidities [13,34,35].

Despite these favorable outcomes, Awake VATS remains a surgical procedure with greater clinical and organizational impact than TBLC, requiring operating room resources, specialized surgical and anesthetic teams, and longer hospitalization. In addition, hospital length of stay associated with Awake VATS is significantly longer than that observed with TBLC, and persistent air leak represents a non-negligible complication that may prolong hospitalization or necessitate discharge with devices such as a Heimlich valve [34,35].

Direct comparison between Awake VATS and TBLC has been reported in a single retrospective study of 132 patients, in which Awake VATS achieved a diagnostic yield of 100% compared with 88.7% for TBLC [22]. While confirming the superior diagnostic accuracy of surgical biopsy, the study also demonstrated significantly lower complication rates such as pneumothorax, shorter hospital stay, and reduced costs with TBLC. These findings indicate that the distinction between the two techniques lies less in diagnostic capability than in procedural burden. Awake VATS provides the highest diagnostic certainty at the expense of greater procedural burden, whereas TBLC achieves diagnosis in most cases with lower procedural impact, underscoring their functional complementarity rather than competition [17,44].

The COLD trial supports a TBLC-first strategy followed by surgical lung biopsy when required, showing that selective surgical escalation can achieve diagnostic accuracy comparable to upfront surgery while reducing complications and hospital stay. However, the trial should not be interpreted as definitive validation of a fixed sequence of TBLC followed specifically by Awake VATS. Rather, it supports the broader principle that TBLC may be used as the initial histological procedure in appropriately selected patients, with subsequent surgical biopsy, either conventional VATS or Awake VATS according to local expertise and patient characteristics, reserved for non-diagnostic or insufficient cases [17,34].

## 6. Critical Synthesis: A New Integrated Diagnostic Model

**Figure 2:**
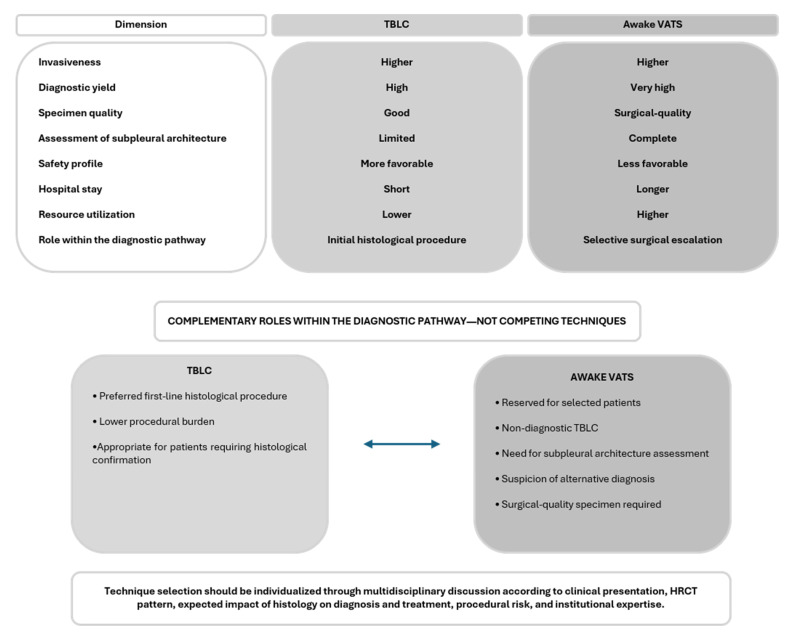
Complementary trade-off between TBLC and Awake VATS within a step-up diagnostic strategy for interstitial lung disease.

Available evidence supports the view that TBLC and Awake video-assisted thoracoscopic surgery should be regarded as complementary rather than competing techniques within a unified diagnostic pathway [11,42] (Figure 2). Awake VATS offers near-complete tissue quality and high diagnostic accuracy but entails a relevant surgical, logistical, and economic burden. TBLC, although associated with a lower diagnostic yield, provides a less invasive option with a superior safety profile and reduced hospitalization and costs [11,17].

Comparative evidence, including data from the only available direct comparison study and the randomized COLD trial, supports a sequential diagnostic strategy, which achieves diagnostic accuracy comparable to upfront surgery while limiting exposure to invasive procedures [17]. In this context, TBLC may represent an appropriate first-line approach for most patients with ILD requiring histological assessment, whereas Awake VATS is best reserved for non-diagnostic cases or for complex clinical–radiological scenarios requiring larger specimens [22].

This integrated, step-up approach reflects a conceptual shift from choosing a single procedure to designing a personalized diagnostic pathway guided by multidisciplinary evaluation, optimizing diagnostic accuracy, patient safety, and healthcare sustainability [6,11,17].

TBLC provides lower invasiveness, lower procedural burden, and good diagnostic performance, whereas Awake VATS provides larger surgical-quality specimens and higher diagnostic yield at the expense of greater resource utilization. Rather than competing techniques, they should be considered complementary procedures selected according to patient characteristics, expected diagnostic benefit, and multidisciplinary evaluation.

## 7. Discussion

Current evidence on lung biopsy techniques in ILD reflects a substantial shift in diagnostic practice. Until around 2010, histological diagnosis relied almost exclusively on surgical biopsy; since then, technological advances and accumulating data have enabled a more flexible, patient-tailored diagnostic approach. This evolution has been gradual and driven by improvements in both procedural safety and clinical decision-making [45,46]. A central finding is that ILD diagnosis cannot be based on a single technique. Rather than identifying an intrinsically superior procedure, the key issue is selecting the most appropriate method for a given clinical scenario. In this framework, TBLC and Awake VATS are complementary tools with distinct roles.

TBLC offers a favorable balance between diagnostic yield and safety, with markedly lower invasiveness than surgery. Technical refinements have improved its reproducibility and reliability, allowing acquisition of larger, higher-quality samples than conventional forceps biopsy while preserving an endoscopic approach. This has reduced the need for upfront surgical biopsy in many patients [18,47,48,49].

Awake VATS, on the other hand, represents an evolution of surgical biopsy toward greater safety and reduced aggressiveness. It provides surgical-quality specimens while avoiding general anesthesia and mechanical ventilation, making it particularly suitable for frail patients or those with limited respiratory reserve [50,51].

Comparative data indicate that Awake VATS achieves higher diagnostic accuracy but at the cost of greater invasiveness, longer hospitalization, and higher resource use, whereas TBLC is less accurate but safer and more sustainable. Accordingly, TBLC is sufficient in many cases, while Awake VATS is reserved for situations in which TBLC is non-diagnostic or inadequate. This complementary approach was supported by the 2024 COLD trial, which showed that a step-up strategy yields diagnostic accuracy comparable to immediate surgery while significantly reducing complications [17]. These findings indicate that histological diagnosis in interstitial lung diseases should be conceptualized not as an “either/or” choice, but as a sequential, two-step pathway in which a minimally invasive approach (TBLC) is followed by surgical biopsy (Awake or conventional VATS) only when necessary (Table 4). This strategy is physiologically sound, clinically efficient, and supported by robust evidence. Importantly, the available evidence should not be interpreted as definitive validation of a fixed sequence of TBLC followed specifically by Awake VATS. Rather, current data support a TBLC-first strategy with selective surgical escalation when histological uncertainty persists, while the choice between Awake VATS and conventional surgical biopsy should be individualized according to patient characteristics, institutional expertise, and available resources.

Moreover, the progressive standardization of TBLC is reducing the inter-center variability that characterized early-generation studies. The introduction of single-use cryoprobes, new probe sizes, electromagnetic navigation techniques, and shared protocols is expected to further narrow the gap between ILD referral centers and emerging centers [18,47,48]. In parallel, advances in anesthesiologic management and sedation techniques are increasing the feasibility of Awake VATS, even in frail patients. Some highly specialized centers have reported simplified anesthesiologic pathways, although these approaches require careful patient selection, dedicated training, and institutional expertise [50,51].

MDD will assume an increasingly central role, not only in diagnostic integration but also in procedural planning, guiding biopsy site selection, risk stratification, interpretation of histological findings, and identification of cases in which surgery can be safely avoided. Within this framework, TBLC should be regarded not as an isolated technique but as part of an integrated diagnostic ecosystem which includes imaging, physiology, pathology, and clinical decision-making [45,52].

Awake VATS remains an important option in a selective subset of patients, including those with predominantly subpleural disease, marked spatial heterogeneity, strong suspicion of alternative diagnoses not adequately assessed by TBLC, or non-diagnostic endoscopic sampling. The complementary use of the two techniques minimizes unnecessary surgical interventions while preserving diagnostic accuracy, aligning ILD diagnostics with principles of personalized medicine and healthcare sustainability [17,46].

The clinical implications of this review are substantial in redefining the role of biopsy methodologies in ILD diagnostics. Using TBLC as a first-line histological procedure may reduce patient morbidity, hospitalization, and costs, an important consideration in resource-constrained healthcare systems. Awake VATS remains essential when TBLC is non-diagnostic or inadequate, providing high-quality surgical specimens while avoiding many risks of conventional VATS under mechanical ventilation. A sequential, step-up strategy limits upfront surgical biopsies to selected cases, aligning ILD diagnosis with principles of personalized medicine. Ongoing standardization and technological advances in TBLC are expected to further improve safety and diagnostic performance even in less specialized centers, facilitating wider adoption and easier access to ILD diagnostics [17,18,46,47,48]. However, successful implementation of this pathway remains highly dependent on institutional expertise, procedural volume, and multidisciplinary experience.

Several limitations should be acknowledged. First, most available TBLC data derive from observational studies, registries, and single-center experiences with heterogeneous patient selection, procedural techniques, sample numbers, probe sizes, anesthesia protocols, and complication definitions [46,49,50,53]. Second, the evidence supporting Awake VATS in ILD remains more limited than that supporting TBLC and is largely based on small series, single-center studies, and selected expert settings. Third, publication bias may favor successful procedural experiences and underestimate complications or conversion rates. Fourth, the external validity of outcomes reported by high-volume ILD referral centers may be limited, particularly in institutions without dedicated MDD, experienced interventional pulmonology, thoracic surgical expertise, anesthesiology support, or intensive-care backup. Finally, although the COLD trial provides important support for a TBLC-first strategy with selective surgical escalation, further multicenter studies are needed to define when Awake VATS, rather than conventional VATS, should be preferred as the surgical step. Centers adopting this pathway should develop standardized protocols, prospective audit systems, predefined complication-management strategies, and clear referral pathways for patients requiring surgical escalation.

Near-term developments most likely to influence clinical practice include further standardization of TBLC protocols, broader use of balloon blockers for bleeding prevention, refinement of cryoprobe size selection, improved biopsy site planning through MDD, and wider availability of navigation-assisted sampling in selected complex cases. For Awake VATS, clinically relevant progress will depend mainly on structured anesthesiology protocols, standardized conversion criteria, improved postoperative air-leak management, and training pathways for thoracic teams [18,47,48,49,50,51].

More experimental developments, including artificial intelligence-assisted radiological targeting, digital pathology support, and AI-guided bronchoscopy, are promising but remain insufficiently validated for routine decision-making in ILD biopsy pathways. These approaches should be presented as investigational tools rather than established components of current practice [54,55].

In this evolving context, the growing complexity of ILD phenotypes will necessitate even closer multidisciplinary integration, with MDD serving not only as a diagnostic forum but also as the cornerstone for selecting the most appropriate biopsy strategy, integrating clinical, radiological, pathological, and procedural considerations within a personalized diagnostic pathway [45,52].

## 8. Conclusions

The histological diagnosis of interstitial lung diseases has evolved substantially over the past decade. TBLC has become a reliable and safe first-line histological technique, providing high-quality tissue with markedly lower invasiveness than surgery, while Awake video-assisted thoracoscopic surgery has refined surgical biopsy by avoiding mechanical ventilation and reducing anesthesiologic risk.

Current evidence supports their integration within a stratified diagnostic pathway, in which TBLC may be used as the initial histological procedure and surgical biopsy is reserved for selected cases when additional tissue is required, achieving high diagnostic accuracy while reducing complications and resource utilization. Ongoing technical and methodological advances are expected to further improve the performance and complementarity of both approaches. However, successful implementation of this strategy depends on multidisciplinary expertise, institutional experience, and careful patient selection.

Contemporary ILD diagnosis therefore requires a multimodal and personalized framework, grounded in MDD, where TBLC and Awake VATS serve as complementary components of a unified diagnostic strategy that optimizes accuracy, safety, and patient-centered care. Selection of the most appropriate technique must be guided by patient-specific clinical, radiological, and functional characteristics and embedded within a structured multidisciplinary pathway. This integrated model has the potential to enable accurate diagnosis while minimizing procedural risk, healthcare resource utilization, and patient burden, thereby improving the overall quality of ILD care.

## Figures and Tables

**Table 1:** Representative studies on TBLC and Awake VATS in ILD: diagnostic yield and safety outcomes.

Study (Year)	Patient (*n*)	Technique	Diagnostic Yield (%)	Safety Outcomes (%)	Key Message
Randomized Controlled Trials					
Bian (2024) [18]	250 (125/125)	TBLC (balloon vs. no balloon)	~80%	Bleeding: moderate 26.4% vs. 6.4% severe 1.6% vs. 0.8%	Balloon pre-placement improves safety without affecting yield
Kalverda (2024) [17]	55 (28/27)	TBLC-first vs. upfront SLB	89% vs. 88%	Serious adverse complications 4% vs. 50% In-hospital stay 1 vs. 5 days	Step-up strategy reduces burden with similar accuracy
Kronborg-White (2025) [19]	33 (15/18)	ENB-TBLC vs. FS-TBLC	93% vs. 61%	Bleeding 66.6% vs. 61.1% Pneumothorax 20% vs. 11.1% Procedure time 62 vs. 44 min Biopsies taken 93% vs. 41% Contribution to the final diagnosis 93% vs. 61%	ENB improves targeting without increasing yield
Prospective Observational Studies					
Ravaglia (2025) [20]	60 (30/30)	TBLC (1.7 mm vs. 1.9 mm)	100% vs. 93.3%	Bleeding 23.3% vs. 30% Pneumothorax 30% vs. 33.3%	Smaller probes improve safety with preserved yield
Zhang (2026) [21]	52	TBLC (1.1 mm cryoprobe)	88.5%	Severe bleeding 3.8% Pneumothorax 1.9%	Mini-probe approach shows favorable safety profile
Retrospective Comparative Studies					
Katgi (2022) [22]	132 (88/44)	TBLC vs. Awake VATS	88.7% vs. 100%	Pneumothorax 6.8% (TBLC) Bleeding (TBLC): mild 61.9% moderate 35.7% severe 2.4% AE-ILD 2.3% (TBLC) Air leak 25% (Awake VATS) In-hospital stay 2 vs. 8 days Costs $172 vs. $516	TBLC less invasive with slightly lower yield
Systematic Reviews and Meta-analyses					
Rodrigues (2022) [23]	4550 (2824/1814)	TBLC vs. SLB	77.1% vs. 95.3 (histology) 76.8% vs. 93.5% (MDD) *	Pneumothorax 9.2% vs. 5.5% AE-ILD 1.4% vs. 2.0% Mortality 0.6% vs. 1.7% Significant bleeding 9.9% (TBLC) Pneumonia 2.1% (SLB) Thoracic pain 3.4% (SLB) Air leak 1.8% (SLB) §	TBLC safer but less accurate than SLB
Patirelis (2024) [24]	675	Awake VATS	85–100%	Overall complication <10% 30-day mortality 0	Awake VATS provides high diagnostic accuracy with reduced perioperative risk

Representative studies selected according to study design, sample size, clinical relevance, and contribution to the proposed diagnostic framework. Studies are grouped by design to facilitate interpretation of the available evidence. Direct comparisons of diagnostic yield and safety outcomes across studies should be interpreted with caution because of heterogeneity in patient populations, procedural techniques, outcome definitions, and safety reporting. * Pooled diagnostic yield for TBLC was derived from 17 studies (histopathology) and 23 studies (MDD), while SLB estimates were based on 8 and 10 studies, respectively. § Complication data were derived from 41 studies. For TBLC, bleeding (26 studies), pneumothorax (30 studies), and acute exacerbation of ILD (18 studies) were reported. For SLB, complications were reported in 13 studies. Thirty-day mortality was reported in 29 studies. Abbreviations: TBLC, transbronchial lung cryobiopsy; Awake VATS, awake video-assisted thoracoscopic surgery; SLB, surgical lung biopsy; ENB, electromagnetic navigation bronchoscopy; FS, fluoroscopy-guided sampling; AE-ILD, acute exacerbation of interstitial lung disease; MDD, multidisciplinary discussion.

**Table 2:** Comparative features of TBLC and Awake VATS in ILD diagnosis.

Feature	TBLC	Awake VATS
Procedural approach	Endoscopic transbronchial sampling using cryoprobe	Surgical thoracoscopic lung biopsy under spontaneous ventilation
Invasiveness	Minimally invasive	Moderately invasive
Anesthesia	Deep sedation/general anesthesia; airway protection often preferred	Locoregional anesthesia with light-to-moderate sedation
Ventilation	Variable depending on the setting	Spontaneous ventilation preserved
Sample/architecture	5–7 mm samples; good tissue architecture	Large surgical specimen; excellent architecture including subpleural tissue
Diagnostic yield	80–81% (>90% in expert centers)	85–100% (near-complete diagnostic accuracy)
Main limitations	Lower yield than surgery; possible non-representative sampling	Grater procedural burden; longer hospitalization
Typical role	First-line histological procedure	Second-line/rescue procedure after non-diagnostic TBLC

Comparison between TBLC and Awake VATS in terms of procedural approach, invasiveness, anesthesia, ventilation strategy, sample characteristics, diagnostic yield, limitations, and typical clinical role. Abbreviations: TBLC: transbronchial lung cryobiopsy; Awake VATS: awake video-assisted thoracoscopic surgery.

**Table 3:** Key determinants of TBLC diagnostic yield and safety.

Domain	Determinant	Impact on Diagnostic Yield and/or Complication Risk
Planning	Pre-procedural MDD target selection	Primarily improves diagnostic yield by increasing specimen representativeness and reducing sampling error
Airway/anesthesia	General anesthesia and balloon blocker	Primarily improves procedural safety through better bleeding control and may enhance specimen quality
Device	Cryoprobe diameter and generation	Influences both diagnostic yield and safety by affecting specimen size, maneuverability, and complication risk
Target choice	Transitional rather than end-stage fibrotic areas	Primarily improves diagnostic yield and histopathological interpretability
Guidance	Electromagnetic navigation	Primarily improves diagnostic yield through more accurate targeting and tissue representativeness; may also improve safety by reducing sampling errors
Center expertise	High-volume expert setting	Improves both diagnostic performance and complication management
Patient-related risk	Advanced fibrosis, low DLCO, pulmonary hypertension	Primarily increases complication risk, particularly pneumothorax, bleeding, and respiratory deterioration

Key determinants influencing the diagnostic yield and safety of TBLC, categorized by procedural domain. The table summarizes the impact of each determinant on diagnostic yield, procedural safety, or both. Abbreviations: MDD, multidisciplinary discussion; DLCO, diffusing capacity of the lung for carbon monoxide.

**Table 4:** Suggested clinical scenarios for TBLC, Awake VATS, or avoidance of biopsy within a step-up diagnostic strategy.

Clinical Situation	Preferred Strategy	Rationale
Indeterminate HRCT pattern requiring histological clarification	TBLC first	Balances diagnostic yield, safety, and procedural burden
Histology expected to influence diagnosis, prognosis, treatment, or eligibility for disease-specific therapy	TBLC first	Provides tissue diagnosis with lower invasiveness than surgery
Preserved or moderately impaired functional reserve and acceptable bronchoscopic risk	TBLC first	Favorable risk–benefit profile
Non-diagnostic or equivocal TBLC	Awake VATS	Completes the diagnostic workup with high accuracy
Need for subpleural architecture assessment or extensive histological characterization	Awake VATS	Provides larger, more representative surgical specimens
Marked radiological heterogeneity or suspicion of alternative diagnoses insufficiently assessed by TBLC	Awake VATS	Surgical-quality tissue may be required
Severe pulmonary hypertension, advanced respiratory failure, major frailty, unstable cardiovascular disease, or extensive comorbidity burden	Avoid biopsy when appropriate	Procedural risk may outweigh potential diagnostic benefit
Histology unlikely to modify diagnosis or management (e.g., high-confidence clinico-radiological diagnosis)	Avoid biopsy when appropriate	Limited expected clinical utility

Proposed clinical decision framework for selecting the most appropriate diagnostic strategy (TBLC, Awake VATS, or avoidance of biopsy) according to clinical scenario, procedural risk, expected impact of histology on diagnosis and treatment decisions, and underlying rationale. Abbreviations: HRCT, high-resolution computed tomography; TBLC, transbronchial lung cryobiopsy; Awake VATS, awake video-assisted thoracoscopic surgery.

## Data Availability

No new data were created or analyzed in this study. Data sharing is not applicable to this article.
